# Pembrolizumab for MSI-high/TMB-high bone and soft tissue sarcomas: a retrospective single-institution study

**DOI:** 10.3389/fonc.2026.1751879

**Published:** 2026-05-11

**Authors:** Tomonori Kawasaki, Tomoaki Torigoe, Daisuke Tsujimoto, Takuya Watanabe, Masataka Hirasaki, Yasuo Kamakura, Masanori Wako, Kouhei Mitsui, Jiro Ichikawa

**Affiliations:** 1 Department of Pathology, Saitama Medical University International Medical Center, Hidaka, Saitama, Japan; 2 Department of Orthopedic Oncology and Surgery, Saitama Medical University International Medical Center, Hidaka, Saitama, Japan; 3 Department of Orthopedic Surgery, Saitama Medical Center, Saitama Medical University, Kawagoe, Japan; 4 Department of Clinical Cancer Genomics, Saitama Medical University International Medical Center, Hidaka, Saitama, Japan; 5 Department of Orthopedic Surgery, Interdisciplinary Graduate School of Medicine, University of Yamanashi, Chuo, Yamanashi, Japan

**Keywords:** checkpoint inhibitors, immunotherapy, microsatellite instability, pembrolizumab, sarcoma, tumor mutational burden

## Abstract

**Introduction:**

Bone and soft tissue sarcomas are rare, heterogeneous malignancies with limited treatment options for advanced stages. Pembrolizumab, an anti-PD-1 immune checkpoint inhibitor (ICI), is approved for tumors exhibiting high microsatellite instability (MSI-high) or high tumor mutation burden (TMB-high); however, its efficacy remains unclear.

**Methods:**

We reviewed records of 40 patients with advanced/recurrent sarcoma who underwent genomic profiling. MSI status and TMB were assessed using the NCC Oncopanel^®^ or FoundationOne^®^. Patients identified as MSI-high and/or TMB-high received pembrolizumab (200 mg/body weight every 3 weeks). Clinical outcomes were evaluated using RECIST v1.1 and adverse events graded per CTCAE v5.0.

**Results:**

Three patients (7.5%) were MSI-high and/or TMB-high: one each with undifferentiated pleomorphic bone sarcoma, leiomyosarcoma, and dedifferentiated chondrosarcoma. All had metastatic disease and received chemotherapy. Pembrolizumab was administered for 5–25 cycles for 5–18 months. Two patients achieved a partial response, and one had stable disease, yielding an objective response rate of 67% and a mean response duration of 12.7 months. No complete response or disease progression occurred. The adverse events were limited to grade 2 thyroid dysfunction and skin toxicity. No grade ≥3 events or treatment discontinuations occurred.

**Conclusion:**

Pembrolizumab demonstrated promising efficacy and tolerability in MSI-high/TMB-high sarcomas. These findings support biomarker-driven immunotherapy in patients with sarcomas. Comprehensive genomic profiling should be considered in refractory cases. Further studies are warranted to validate these results and explore additional biomarkers.

## 1 Introduction

Bone and soft tissue sarcomas are rare, heterogeneous malignancies arising from mesenchymal tissues, accounting for less than 1% of all adult cancers (1). Despite advances in surgical techniques and multimodal therapies, the prognosis for patients with advanced or recurrent sarcomas remains poor (2). Standard chemotherapy regimens, such as doxorubicin-based combinations, offer limited efficacy, and there is a pressing need for novel therapeutic strategies (3).

Immune checkpoint inhibitors (ICIs), particularly those targeting the PD-1/PD-L1 axis, have revolutionized the treatment of various solid tumors (4). Pembrolizumab, an anti-PD-1 monoclonal antibody, received tissue-agnostic approval in December 2018 for the treatment of unresectable or metastatic solid tumors characterized by high microsatellite instability (MSI-high) or high tumor mutation burden (TMB-high) (5, 6). These biomarkers are associated with an increased neoantigen load and enhanced immunogenicity, rendering tumors more susceptible to immune-mediated destruction (6).

Although the efficacy of pembrolizumab has been demonstrated in malignancies, such as colorectal, endometrial, and gastric cancers (7), its role in sarcomas remains unclear. Sarcomas are generally considered immunologically “cold” tumors, with low mutational burden and limited immune infiltration (8). However, emerging evidence suggests that a subset of sarcomas harboring MSI-high or TMB-high profiles may benefit from ICI therapy (9). Importantly, TMB varies substantially across sarcoma subtypes: undifferentiated pleomorphic sarcoma (UPS) and osteosarcoma often exhibit intermediate-to-high TMB, whereas leiomyosarcoma, liposarcoma, and chondrosarcoma typically show low TMB (10). MSI−high status is also rare, occurring in only 1–5% of sarcomas. Moreover, although TMB−high/MSI−high tumors may respond to ICIs, these biomarkers lack sensitivity, and responses have been reported even in TMB−low sarcomas (11, 12). Thus, no gold−standard biomarker for ICI responsiveness in sarcoma has been established. This retrospective study aimed to evaluate the clinical outcomes of pembrolizumab treatment in patients with advanced bone and soft tissue sarcomas identified as MSI-high and/or TMB-high by genomic profiling at a single institution.

## 2 Methods

### 2.1 Patient selection

We retrospectively reviewed the records of 40 patients with advanced or recurrent bone and soft tissue sarcomas, who underwent comprehensive genomic profiling at our institution between October 2019 and November 2023. All patients had exhausted standard chemotherapy options and were considered for immunotherapy based on their molecular eligibility. This study was conducted in accordance with the Declaration of Helsinki and was approved by the Ethics Committees of Saitama Medical University International Medical Center. Written informed consent was obtained from all participants.

### 2.2 Genomic profiling

Genomic testing was performed using either the NCC Oncopanel^®^ with MSI testing (n=8) or FoundationOne^®^ (n=32). All TMB analyzes were performed on tumor tissue samples. The MSI status was determined using polymerase chain reaction-based or next-generation sequencing methods, whereas TMB was quantified as the number of somatic mutations per megabase (mut/Mb). TMB-high was defined as ≥10 mut/Mb.

### 2.3 Treatment protocol

Patients identified as MSI-high and/or TMB-high were administered pembrolizumab at a fixed dose of 200 mg/body every 3 weeks. The treatment was continued until disease progression, unacceptable toxicity, or patient withdrawal. Radiological assessments were performed every 8–12 weeks using RECIST v1.1 criteria. Adverse events were graded according to the Common Terminology Criteria for Adverse Events (CTCAE) version 5.0 (Japan Clinical Oncology Group Japanese translation).

### 2.4 Data collection and analysis

Clinical data, including demographics, histopathological diagnosis, prior treatments, genomic findings, treatment duration, response, and survival outcomes, were extracted from electronic medical records. Descriptive statistics were used to summarize the patient characteristics and treatment outcomes. Radiologic and toxicity assessments were performed as part of routine clinical care, and RECIST v1.1 and CTCAE v5.0 evaluations for this study were conducted retrospectively during chart review.

## 3 Results

### 3.1 Genomic findings

Among the 40 patients profiled, three (7.5%) were identified as MSI-high and/or TMB-high. Two patients were MSI-high, and three were TMB-high, including two patients with MSI-high and TMB-high tumors, whereas one patient was TMB-high only. These findings are consistent with previously reported prevalence rates in sarcomas, which range from 2.8% to 5% for MSI-high and 1.7% to 9.4% for TMB-high.

### 3.2 Patient characteristics

Patient characteristics were shown in **Table 1**. The three patients treated with pembrolizumab included two males and one female, aged 51–71 years. The histopathological diagnoses included UPS of the bone (n=1), leiomyosarcoma (n=1), and dedifferentiated chondrosarcoma (n=1). All patients had metastatic disease involving the lungs and/or bones and had previously received chemotherapy, including doxorubicin-based regimens.

**Table 1:** Characteristics of MSI-high and/or TMB-high cases.

Characteristic	Case 1	Case 2	Case 3
Age (years)	54	71	51
Sex	Male	Female	Male
Diagnosis	Undifferentiated pleomorphic sarcoma of bone	Leiomyosarcoma	Dedifferentiated chondrosarcoma
Primary Site	Left pubis	Retroperitoneum	Pelvis
Metastatic Sites	Bone, lung	Lung	Lung
Prior Chemotherapy	Doxorubicin + Cisplatin, Ifosfamide	Doxorubicin-based	Doxorubicin-based
MSI Status	High	High	Low
TMB (mutations/Mb)	21	12	11
ECOG Performance Status	1	1	0

MSI, microsatellite instability; TMB, tumor mutation burden; ECOG, Eastern Cooperative Oncology Group.

### 3.3 Treatment outcomes

Pembrolizumab was administered for 5–25 cycles (mean: 15 cycles). The treatment duration ranged from 5 to 18 months (mean: 12.7 months). Response evaluation revealed partial response (PR) in two patients, stable disease (SD) in one patient, and an objective response rate (ORR) of 67%. No cases of complete response or progressive disease during treatment.

### 3.4 Adverse events

Treatment-related adverse events were limited and manageable. Grade 2 thyroid dysfunction (n=1) and Grade 2 skin toxicity (n=1) were observed. No grade ≥3 adverse events or treatment discontinuations attributable to toxicity occurred.

### 3.5 Survival analysis

Given the extremely small sample size (n=3), survival outcomes were descriptive only. At the time of analysis, all three patients were alive, and the median overall survival had not been reached. Although the median overall survival of the non−ICI cohort was 11.2 months, this value is provided only as contextual reference, as direct comparison was not feasible because of heterogeneity in histology and treatment background.

### 3.6 PD−L1 expression

PD−L1 immunohistochemistry was not uniformly performed across the cohort. PD−L1 status was unavailable for three patients. This limitation restricts the interpretation of PD−L1 as a predictive biomarker in this study.

### 3.7 Case highlight

**Figure 1:**
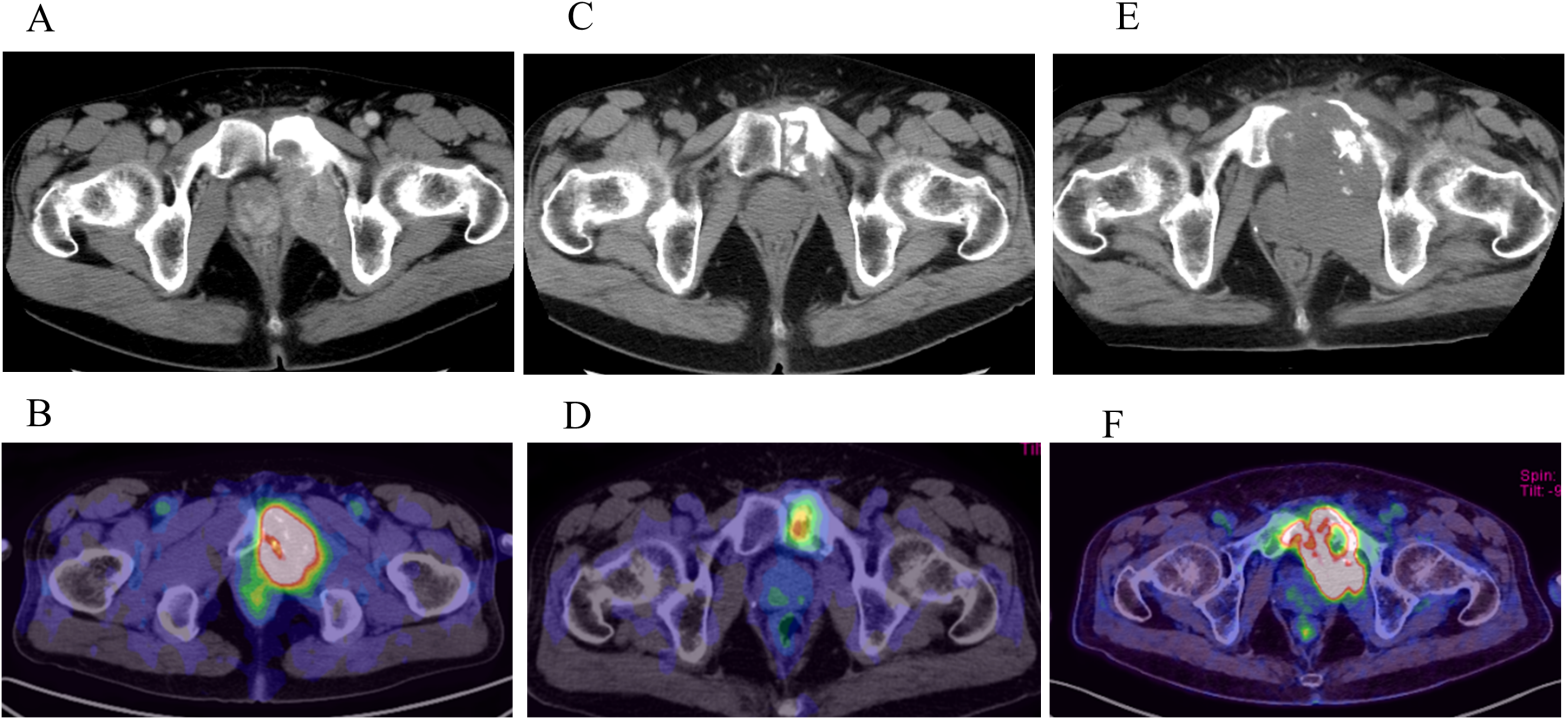
Imaging-based assessment of treatment response over time: Pretreat; computed tomography (CT) **(A)** and positron emission tomography (PET)-CT **(B)** 12 months after initiation of pembrolizumab treatment; CT **(C)** and PET-CT **(D)** 18 months; CT **(E)** and PET-CT **(F)**.

A 54-year-old man with UPS of the left pubis and multiple bone metastases (involving the ilium, sacrum, and ribs) presented with progressive pain and was referred to our hospital. Initial chemotherapy with doxorubicin and cisplatin was followed by ifosfamide in response to renal dysfunction. Disease progression was observed in patients with multiple pulmonary metastases. Genomic profiling revealed an MSI-high status and TMB of 21 mutations/Mb. Although marked tumor shrinkage was achieved 12 months after pembrolizumab treatment, tumor regrowth was observed at 18 months (**Figure 1**).

## 4 Discussion

ICIs have transformed the therapeutic landscape for many solid tumors, particularly those with MSI-high or elevated TMB-high (4, 6). These biomarkers are associated with increased neoantigen presentation and enhanced immunogenicity, rendering tumors more susceptible to immune-mediated destruction (6). Pembrolizumab, an anti-PD-1 monoclonal antibody, has received tissue-agnostic approval for MSI-high/TMB-high refractory solid tumors (5). However, its role in bone and soft tissue sarcomas remains underexplored, owing to the rarity and molecular heterogeneity of these malignancies (8).

In our retrospective study, three of the 40 patients (7.5%) with advanced sarcomas were identified as MSI-high and/or TMB-high. This prevalence aligns with previous reports, which estimated MSI-high in 2.8–5% and TMB-high in 1.7–9.4% of sarcomas (9). Despite the low frequency, the observed ORR of 67% and mean response duration of 12.7 months were clinically meaningful. Two patients achieved PRs, and one maintained SD with no progression during pembrolizumab therapy. Notably, although limited by the extremely small sample size, the observed ORR of 67% appears higher than that reported in unselected sarcoma cohorts. In the SARC028 trial, pembrolizumab demonstrated an ORR of 23% in UPS, 10% in liposarcoma, and only 5% in bone sarcomas (13), highlighting the potential value of biomarker−driven selection. Similarly, in the KEYNOTE−158 study, the ORR among patients with TMB−high sarcomas was approximately 20–25% in small cohorts (14), which is notably lower than the 67% ORR observed in our cohort. Although cross−study comparisons should be interpreted with caution given the small sample size, these findings suggest that MSI−high/TMB−high status may enrich for patients more likely to benefit from pembrolizumab.

The AcSé Pembrolizumab study further supports the use of pembrolizumab in rare tumors, including sarcomas, where responses were enriched among tumors harboring actionable immune−related biomarkers (15). However, it is important to note that TMB and MSI lack sensitivity as predictive biomarkers in sarcoma. Pan−sarcoma analyzes, including KEYNOTE−158 (14) and A091401 (11), have shown higher response rates among TMB−high tumors; however, responses have also been observed in TMB−low sarcomas (12). Although TMB−high/MSI−high status may identify a subset of patients more likely to respond, these markers do not capture all responders, and no gold−standard biomarker has been established.

Recent pan-tumor analyzes, including KEYNOTE−158, support the efficacy of pembrolizumab in MSI-high/TMB-high tumors, including rare histologic subtypes (14). Furthermore, real-world analyzes suggest that tertiary lymphoid structure (TLS) status may influence outcomes in high TMB or MSI-high tumors treated with ICIs (16). Although TLS was not assessed in our cohort, its potential as a complementary biomarker warrants further investigation.

The safety profile of pembrolizumab in our study was favorable, with only grade 2 thyroid dysfunction and skin toxicity observed. No grade ≥3 adverse events or treatment discontinuations occurred. This tolerability is consistent with the broader ICI experience and supports its use in heavily pretreated patients (4).

This study has some limitations. The extremely small sample size (n=3) severely limits statistical interpretation and generalizability. The retrospective design, heterogeneity of histologic subtypes, and variability in prior treatments introduce potential confounding factors. PD−L1 status was unavailable for most cases, and TLSs were not assessed. Additionally, TMB and MSI exhibit substantial heterogeneity across sarcoma subtypes, and their rarity limits broader applicability (10).

Despite these limitations, our findings contribute to the growing body of evidence supporting biomarker-driven immunotherapy for sarcomas. Comprehensive genomic profiling should be considered in patients with advanced disease, particularly when standard options are exhausted. As sequencing technologies become more accessible, identifying actionable biomarkers, such as MSI and TMB, may help personalize treatment and improve outcomes.

## 5 Conclusion

Pembrolizumab demonstrated promising efficacy and tolerability in MSI-high/TMB-high bone and soft tissue sarcomas. Prospective multicenter studies are needed to validate these findings and explore additional biomarkers such as TLS and PD-L1 expression.

## Data Availability

The original contributions presented in the study are included in the article/supplementary material. Further inquiries can be directed to the corresponding author.
